# Respiratory epithelial cells as central mediators of immune crosstalk in SARS-CoV-2 infection

**DOI:** 10.3389/fimmu.2026.1799580

**Published:** 2026-04-17

**Authors:** Xingjian Liu, Zhefen Mai, Lingbin Sun, Liqiang Deng, Moushen Tang, Geng Li, Xiaoyi Yang

**Affiliations:** 1 Shenzhen Hospital (Futian) of Guangzhou University of Chinese Medicine, Shenzhen, China; 2 Chinese Medicine Guangdong Laboratory, Zhuhai, China; 3 School of Pharmaceutical Sciences, Sun Yat-sen University, Guangzhou, China; 4 Guangzhou Laboratory, Guangzhou, China; 5 Science and Technology Innovation Center, Guangzhou University of Chinese Medicine, Guangzhou, China

**Keywords:** COVID-19 pathogenesis, immune crosstalk, immunopathology, respiratory epithelial cells, SARS-CoV-2

## Abstract

Severe acute respiratory syndrome coronavirus 2 (SARS-CoV-2) induces life-threatening acute lung injury (ALI) and disrupts immune homeostasis, however, the role of epithelial-immune cell crosstalk in driving this pathology remains incompletely elucidated. Respiratory epithelial cells (RECs) as the primary targets of SARS-CoV-2 via the ACE2 receptor, act as central mediators of immune crosstalk that balances antiviral defense and immunopathology in COVID-19. Beyond forming a physical barrier against pathogen invasion, RECs regulate bidirectional crosstalk with immune cells (including alveolar macrophages, dendritic cells, neutrophils, and lymphocytes) through multiple mechanisms, such as cytokine signaling, antigen presentation, PD-L1 checkpoint modulation, and renin-angiotensin-aldosterone system (RAAS) dysregulation. Under physiological conditions, these interactions promote viral clearance and epithelial repair; In contrast, dysregulation of such crosstalk leads to excessive inflammatory responses like cytokine storm and impaires tissue regeneration. Elucidating the molecular dynamics underlying REC-immune crosstalk is crucial for gaining insights into the development of targeted therapies (e.g., modulating cytokine signaling, restoring RAAS balance) to mitigate the severity of COVID-19. This review summarized recent findings to clarify how REC-mediated immune crosstalk dictates antiviral responses and pathological outcomes, thereby providing a theoretical basis for optimizing therapeutic strategies that strengthen antiviral immunity while minimizing immunopathology.

## Introduction

The respiratory epithelium in the nasal cavity, trachea, and bronchi is composed of pseudostratified columnar epithelium cells. It then transitions to columnar and cuboidal cells in the bronchioles, and in the alveoli, it forms a thin, single-layered alveolar epithelium. The bronchial and alveolar epithelia are composed of distinct cell populations. Specifically, bronchial epithelial cells include basal cells, ciliated cells, goblet cells, club cells, tuft cells, neuroendocrine cells, and ionocytes (1), whereas the alveolar epithelium primarily comprises type 1 (AT1) and type 2 (AT2) alveolar epithelial cells (AECs) (2).

Given that respiratory epithelial cells (RECs) cover a large surface area in the body, they are prime targets for diverse pathogens, including respiratory viruses that cause illnesses ranging from mild upper respiratory infections to severe diseases, such as bronchiolitis, pneumonia, and acute respiratory distress syndrome (ARDS). Epidemiologically, viruses are the most frequently detected pathogens in adults with community-acquired pneumonia. Among these, rhinoviruses and influenza viruses are the most common, while other aetiologies include human metapneumovirus, respiratory syncytial virus (RSV), parainfluenza viruses, and coronaviruses. Children typically experience annual episodes of respiratory viral infections (3–5). A critical determinant of disease outcome may be the balance between effective antiviral immunity, which limits viral spread and promotes clearance and the prevention of excessive immune-mediated pathology. The respiratory epithelium plays a pivotal role in maintaining this equilibrium (6).

Among respiratory viruses, Severe acute respiratory syndrome coronavirus 2 (SARS-CoV-2), which causes coronavirus disease 2019 (COVID-19), remains a major global concern. According to World Health Organization (WHO) surveillance data, over 7 billion cumulative COVID-19 cases and 7.2 million deaths have been reported worldwide. Variant dynamics included the variant of interest JN.1 (16.3% of sequences in week 5, 2025), decreased prevalence of the variant under monitoring XEC (42.7%), and increased frequency of LP.8.1 (13.9%) and LB.1 (1.2%). Hospitalizations and ICU admissions decreased by 40% and 31%, respectively, among consistent reporters (7). Notably, JN.1 has become a dominant variant of interest due to its enhanced transmissibility, and the declining prevalence of XEC may be associated with improved population immunity. Although hospitalization and ICU admission rates have decreased, the increased number of deaths indicates that vulnerable populations (e.g., older adults and those with underlying diseases) still face a high risk of severe illness. These divergent trends highlight the continuing importance of surveillance, particularly given that the differences in viral variant dynamics and clinical outcomes are closely related to the ability of the respiratory epithelium to recognize different variants and the intensity of immune activation it mediates, further emphasizing the necessity of in-depth research into the immune regulatory mechanisms mediated by respiratory epithelial cells.

During SARS-CoV-2 infection, epithelial cells, including those of the bronchial and nasal mucosa, are the primary viral targets and engage in intricate crosstalk with immune cells, such as neutrophils, macrophages, and monocytes, via molecular interactions and cytokine signaling cascades. These cells express high levels of viral entry factors (angiotensin-converting enzyme 2 [ACE2] and transmembrane serine protease 2 [TMPRSS2]) and CD147 (BSG) with its ligands (PPIA/PPIB and S100A9); binding of these ligands to monocyte/macrophage CD147 enhances immune activation and proinflammatory cytokine release (8). In addition to entry facilitation, epithelial cells recruit and activate immune cells through cytokine secretion. For example, when stimulated by viral mimetics such as Toll-like receptor (TLR) 3 agonists, nasal (HNEpC) and small airway (SAEpC) epithelial cells release TNF-α and CXC chemokine ligand 10 (CXCL10), which recruit monocytes that subsequently differentiate into macrophages. These inflammatory signals also induce macrophage TNF-α and IL-1β secretion (9). In addition, epithelial cells, neutrophils, and monocytes contribute to coagulation dysregulation. Lung epithelial cells release tissue factor (TF) positive extracellular vesicles (EVs), monocytes express TF in platelet-monocyte aggregates, and neutrophils release TF via neutrophil extracellular traps (NETs). Together, these pathways synergistically activate coagulation, promote thrombosis, and correlated with elevated plasma D-dimer levels, disease severity, and mortality (10).

As highlighted above, RECs serve not only as primary viral targets but also as active participants in immune regulation. They mediate crosstalk with diverse immune populations through molecular signaling and cytokine secretion, regulate innate antiviral responses, and contribute to pathological processes, such as coagulation activation via TF-positive EVs. Beyond these roles, infected epithelial cells may initiate intrinsic antiviral defences, orchestrate activation of myeloid and lymphoid cells, and support epithelial tissue repair. These functions are central to balancing viral clearance with the prevention of immune-mediated pathology. In this review, we have collated recent studies that elucidated the multifaceted crosstalk between RECs and immune and non-immune cells (macrophages, dendritic Cells, neutrophils, lymphocytes, and lung-resident immune and stromal cells) during SARS-CoV-2 infection. We also discuss how these crosstalk processes shape immune responses, pathological outcomes (such as thrombosis), and epithelial repair, aiming to provide a theoretical basis for the development of therapeutic strategies targeting REC-related crosstalk pathways.

## Molecular pathogenesis of SARS-CoV-2

Over the past two decades, three zoonotic coronaviruses—SARS-CoV, Middle East respiratory syndrome coronavirus (MERS-CoV), and SARS-CoV-2—have merged (11–13). Unlike endemic coronaviruses, these three zoonotic viruses can replicate in the lower respiratory tract and may induce life-threatening ARDS (14).

**Figure 1:**
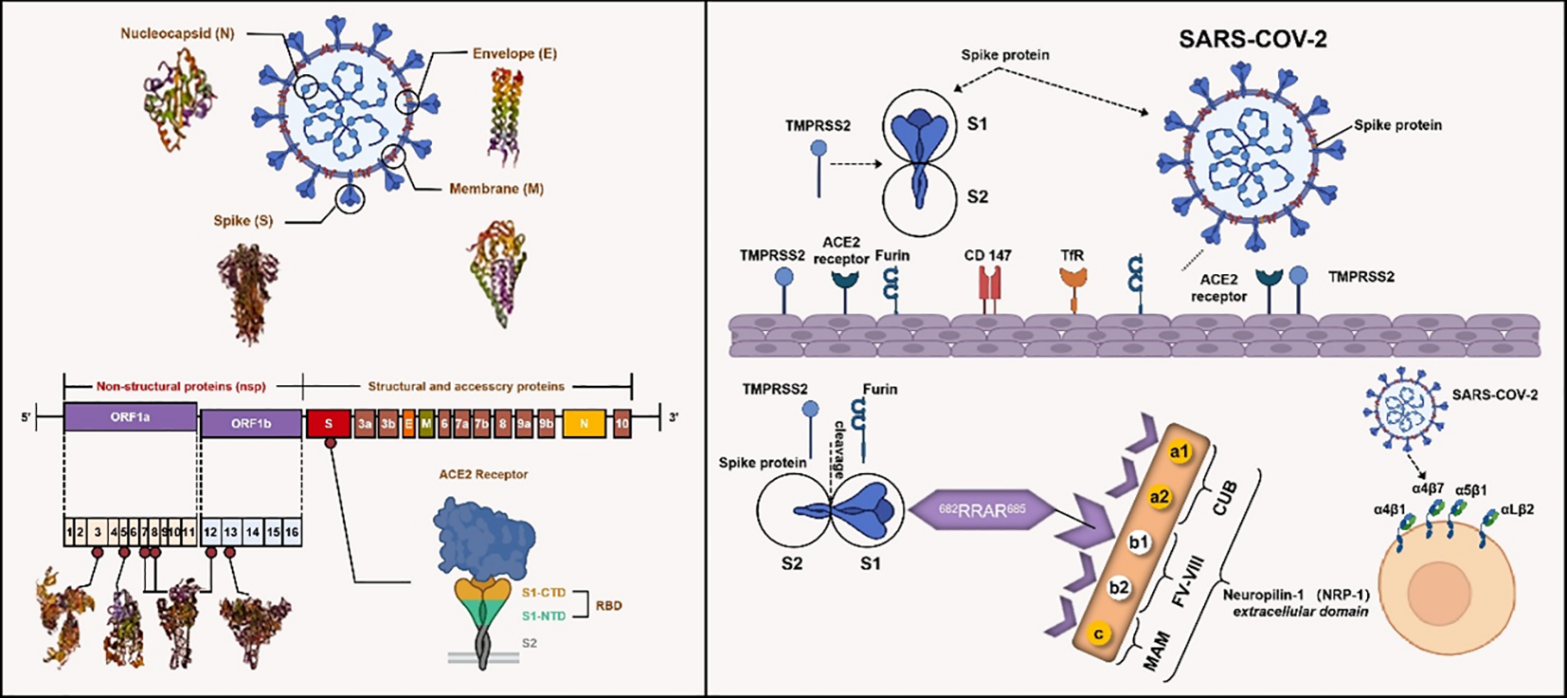
The structural composition, genomic organization, and host cell entry mechanisms of coronaviruses, particularly SARS-CoV-2. SARS-CoV-2 comprises key structural proteins: nucleocapsid protein (N), envelope protein (E), membrane protein (M), and spike protein (S). Its viral genome is divided into regions encoding non-structural proteins (nsps, including ORF1a and ORF1b with nsp1 to nsp16) and structural/accessory proteins. The spike protein of SARS-CoV-2, composed of S1 and S2 subunits, binds to the ACE2 receptor on the host cell membrane with the participation of proteases such as TMPRSS2 and furin, thereby enabling the virus to enter host cells. In addition, studies have found that molecules like CD147, Neuropilin-1 (NRP1), integrins (α4β1, α4β7, a5b1, αLβ2), and TfR also interact with SARS-CoV-2 to mediate viral entry.

SARS-CoV-2 was first detected in Wuhan, China, in late 2019 within of patients with pneumonia. SARS-CoV-2 has approximately 79% genomic nucleotide sequence similarity to SARS-CoV and belongs to the genus *Sarbecovirus* within the family *Coronaviridae* (15). Its genome encodes three classes of proteins: structural proteins (membrane [M] protein, nucleocapsid [N] protein, envelope [E] protein, and spike [S] glycoprotein]); non-structural proteins, most of which constitute the viral replication and transcription complex; and accessory proteins. Structural proteins, together with a host-derived lipid bilayer, form an enveloped virion that delivers viral genomic RNA into host cells. Accessory proteins are dispensable for viral replication but often contribute to immune evasion (16, 17). The S glycoprotein, which assembles into trimers on the virion surface, is the primary determinant of coronavirus infection (18). The S protein is composed of two functional subunits. The S1 subunit binds to the host entry receptor ACE2 (19), while the S2 subunit mediates viral-host membrane fusion. These subunits are separated by the S1–S2 cleavage site, which contains a furin recognition motif and is cleaved by furin in virus-producing cells. Following ACE2 binding on target cells, the S protein undergoes a second cleavage at the S2′site by the TMPRSS2 (20, 21). This cleavage activates the S2 subunit trimer to fuse viral and host lipid bilayers, releasing the viral ribonucleoprotein complex into the cytoplasm. SARS-CoV-2 can also enter via an endosomal route, in which cathepsins mediate S protein cleavage to enable viral internalization. However, this pathway is inefficient in primary epithelial cells, possibly due to the low expression of cathepsins in these cells (22–24). Additional co-receptors (e.g., Neuropilin1) and proteases (e.g., cathepsin L, TMPRSS11D, and TMPRSS13) have been implicated in viral entry (25, 26), but their precise roles in SARS-CoV-2 pathogenesis remain unclear (27) (**Figure 1**).

## Epithelial cell responses to SARS-CoV-2 infections

SARS-CoV-2 enters host cells by binding its surface S protein to the ACE2 receptor on RECs. ACE2 is highly expressed in epithelial populations, including ciliated and club cells in the nasal cavity and bronchi. These cells serve as the primary targets for viral invasion, in which SARS-CoV-2 replicates, induces cellular damage, and impairs epithelial function (28). Following infection, the number of surface cilia on these cells is reduced, compromising mucociliary clearance and promoting the accumulation of pathogens and particulate matter, thereby increasing susceptibility to secondary infections. Additionally, SARS-CoV-2 disrupts intercellular tight junctions, weakening the epithelial barrier and rendering the lungs more vulnerable to invasion by other pathogens. Upon viral entry and replication, infected nasal epithelial cells upregulate the expression of innate immune sensors (TLR7 and RIG-I), effector molecules (type I and III interferons [IFNs]), and pro-inflammatory chemokines (CXCL9, CXCL10, CXCL11, and CCL5) (29). Elevated expression of IL-6, IL-6R, and IL-6ST in lung epithelial cells suggests that SARS-CoV-2 infection may trigger uncontrolled cytokine production, which is—consistent with clinical reports of elevated IL-6 levels in patients with severe COVID-19 (30).

As the infection progresses, SARS-CoV-2 damages alveolar cells, particularly AT2 cells— the sole source of pulmonary surfactant (a lipid-protein complex critical for reducing alveolar surface tension and maintaining lung compliance) (31, 32). AT2 cells represent a major target of SARS-CoV-2. Upon infection, the virus induces AT2 cell dysfunction via multiple interconnected pathways, including direct cytolysis and attack by excessive pro-inflammatory cytokines (such as IL-6 and TNF-α) (33, 34), and endoplasmic reticulum (ER) stress-driven activation of the unfolded protein response (UPR) via PERK and IRE1α pathways (31). This leads to downregulation of surfactant protein genes (Sftpb, Sftpc), marked reductions in hydrophobic surfactant proteins (SP-B, SP-C), surfactant dysfunction with increased surface tension, and emergence of a reprogrammed Krt8+/Cldn4+ transitional AT2 state—all of which compromise alveolar stability (31, 35). Concurrently, AT2 cell injury and lysis cause leakage of cellular contents (DAMPs, viral particles) into the alveolar-capillary interface (33, 36), disrupting the surfactant layer, triggering neutrophil/monocyte recruitment, and promoting neutrophil extracellular trap (NET) formation, which further exacerbates alveolar-capillary barrier damage and vascular leakage (37, 38). The combined effects of surfactant loss/dysfunction and barrier disruption lead to alveolar edema, collapse, and reduced lung compliance, which correlates with progressive respiratory failure in clinical settings (33, 39). Clinical studies confirm that plasma SP-A levels (a surrogate for alveolar-capillary leakage) are significantly elevated in severe COVID-19 (optimal cutoff: 10 ng/mL), while synthetic SP-B peptide mimics (e.g., SMB, B-YL) can bind ACE2 to compete with SARS-CoV-2 spike protein, highlighting therapeutic potential (40, 41). Collectively, this pathological cascade may be a key driver of COVID-19 severity.

**Figure 2:**
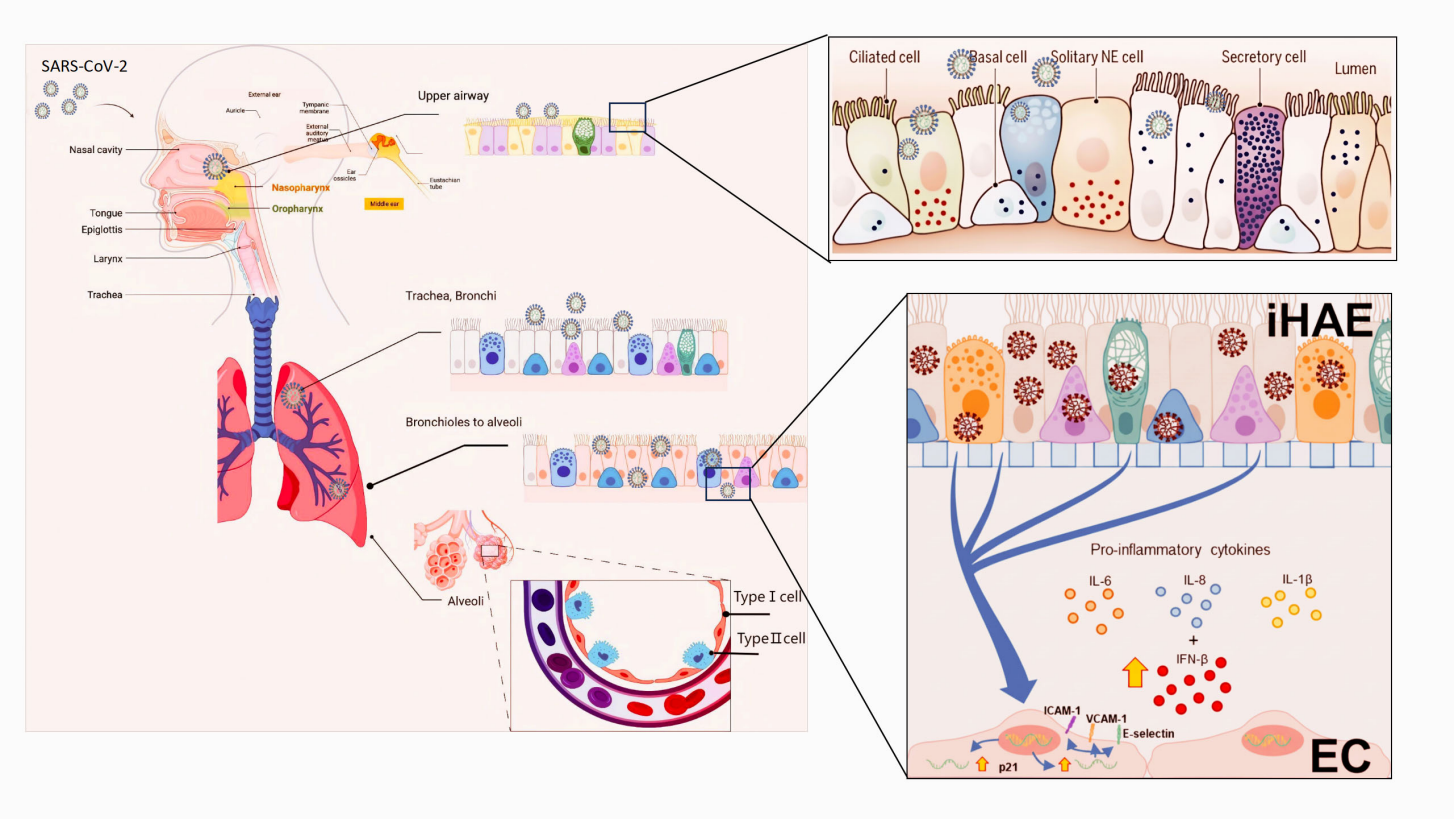
The tropism and pathogenic mechanisms of SARS-CoV-2 in the human airway. The human respiratory system has an anatomical structure comprising the nasal cavity, pharynx, larynx, trachea, bronchi, bronchioles, and alveoli. SARS-CoV-2 enters the respiratory system via the respiratory tract and infects ciliated cells, basal cells, neuroendocrine (NE) cells, and secretory cells within the airway lumen. Subsequently, the infected cells release pro-inflammatory cytokines (IL-6, IL-8, IL-1β) and type I interferons (IFN-α, β). Meanwhile, the upregulation of adhesion molecules (ICAM-1, VCAM-1, E-selectin) and the cell cycle regulator p21 in endothelial cells (EC) triggers inflammatory and immunomodulatory responses, leading to damage to the adhesive junctions of pulmonary blood vessels.

Following epithelial injury, the repair capacity of RECs is impaired, likely due to persistent viral replication or excessive inflammation, which may predispose patients to chronic lung disease. During infection, RECs function as key intermediaries that communicate with other epithelial cells but also engage directly with immune cells. Through cytokine release, they regulate immune cell activation and migration, thereby modulating the local immune response, which is critical for viral clearance and disease progression (**Figure 2**). The effects of SARS-CoV-2 on RECs extend beyond the lungs: systemic inflammatory responses induced by infection can lead to multi-organ dysfunction and severe complications, including ARDS and multi-organ failure (39, 42).

Gene expression profiling of RECs in *in vitro* has provided important insights into their antiviral responses. However, relatively few studies have examined how these *in vitro* findings translate to intact lungs. Notably, significant enrichment of ACE2+/TMPRSS2+ double-positive cells has been observed in primary human bronchial epithelial cells (HBECs) (43). Therefore, bronchial epithelial cells are not the only core target cells for SARS-CoV-2 invasion, but also major contributors to the initiation of the immune-inflammatory response.

## Epithelium/macrophage communication

Resident alveolar macrophages (AMs) are non-migratory cells that maintain alveolar homeostasis through direct interactions with the alveolar epithelium. Bidirectional communication between AMs and AECs occurs through cell surface receptors, gap junctional channels, exchange of secreted microparticles, and cytokine signaling (44–46). Under homeostatic conditions, epithelial cells release inhibitory signals that may contribute to prevent excessive AM activation in airspaces. However, during viral infection, this anti-inflammatory signaling may be suppressed, triggering antiviral immune responses (46, 47).

**Figure 3:**
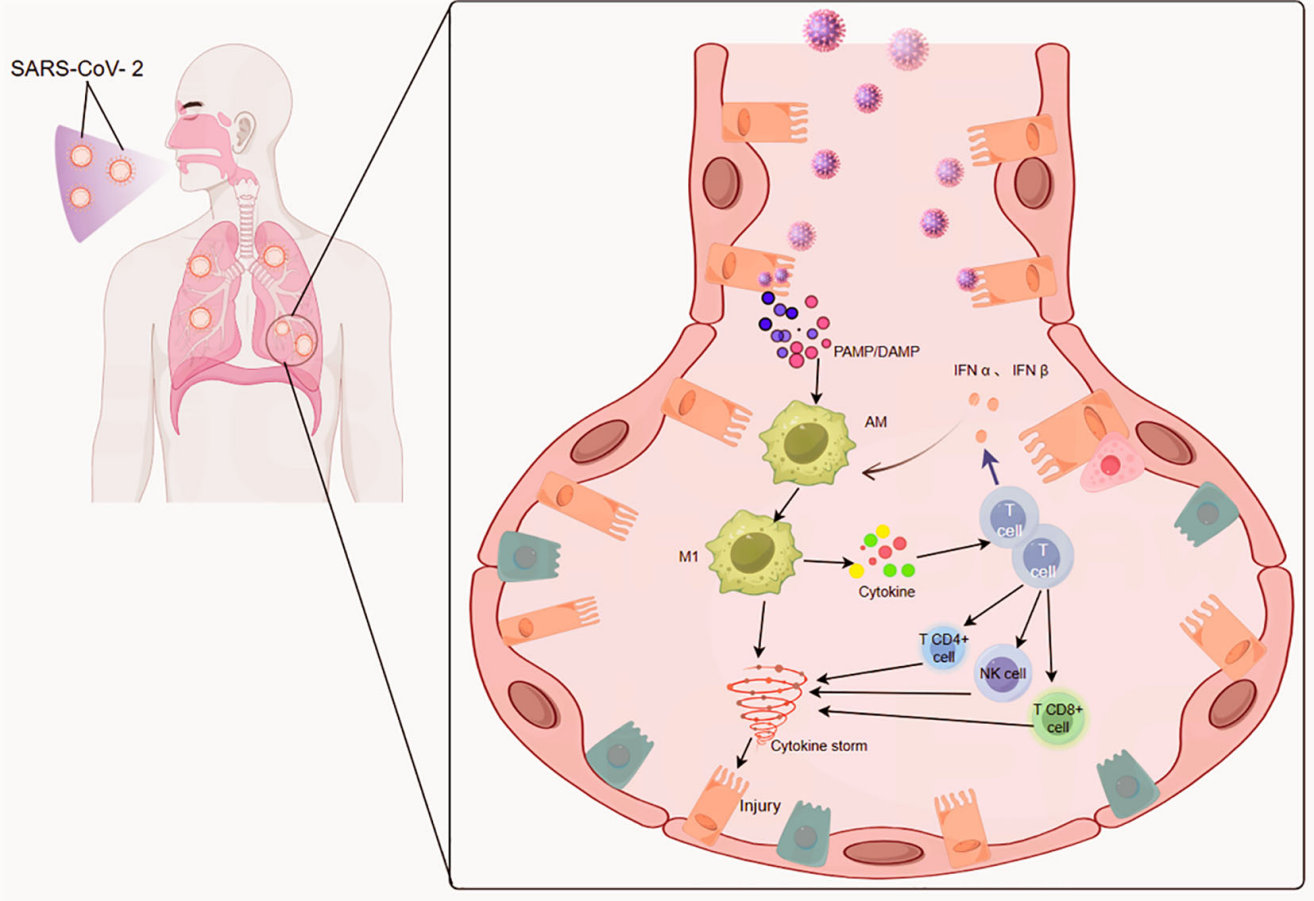
Schematic illustration of epithelium-macrophage communication in the respiratory tract during SARS-CoV-2 infection. SARS-CoV-2 invades respiratory epithelial cells, which subsequently release pathogen-associated molecular patterns (PAMPs) (derived from the virus) and damage-associated molecular patterns (DAMPs) (from injured cells). These mediators recruit and activate alveolar macrophages (AMs) in the local microenvironment. Activated AMs can differentiate into proinflammatory M1-phenotype macrophages, or directly secrete cytokines (e.g., IFNα, IFNβ); these soluble factors further drive the activation and recruitment of adaptive immune cells (CD4+ T cells, CD8+ T cells) and innate immune cells (natural killer [NK] cells). Crosstalk between M1 macrophages and the recruited immune cells amplifies the secretion of proinflammatory mediators, ultimately triggering a dysregulated cytokine storm. This excessive inflammatory response induces direct injury to respiratory epithelial cells, exacerbating the pathological alterations associated with SARS-CoV-2 infection.

As the first line of defence against alveolar pathogens, AMs display high phenotypic plasticity, which allow them to transition between functional states in response to infection. Previous studies have delineated that M1-polarized AMs have an acidic endosomal pH, which facilitates SARS-CoV-2 escape from endosomes into the cytosol to initiate replication (48). In contrast, M2-polarized AMs exhibit a high endosomal pH, which impairs viral escape and promotes lysosomal degradation of the virus (49). Given that the respiratory tract is a mucosa-associated open system, AMs possess high phenotypic plasticity, allowing them to switch from an anti-inflammatory M2 state to a pro-inflammatory M1 state in response to pathogenic invasion (50). By recognizing pathogen-associated molecular patterns (PAMPs) via pattern recognition receptors such as TLRs and NOD-like receptors, AMs rapidly activate NF-κB and MAPK pathways, triggering inflammatory responses. Thus, AMs function as sentinels that initate antiviral immunity. Importantly, the polarization state of AMs profoundly modulates their ability to control SARS-CoV-2 infection (49, 51, 52); SARS-CoV-2 may preferentially replicate in M1 AMs, as M2 AMs mediate early viral control (**Figure 3**).

Transcriptomic profiling has revealed that SARS-CoV-2 infects AMs, which subsequently secrete T cell chemo attractants (53). In turn, recruited T cells release IFN-γ, which induces AMs to produce additional pro-inflammatory cytokines, thereby amplifying T cell activation and creating a positive feedback loop that escalates the local immune response. In most patients with SARS-CoV-2 infection, the alveolar space becomes persistently enriched with T cells and monocytes, a phenomenon linked to dysregulated crosstalk between AMs and adaptive immune cells (53). In addition to resident AMs, monocyte-derived macrophages are recruited during viral infection. For instance, in RSV infection, HBECs upregulate chemokines, such as regulated on activation normal T cell expressed and secreted (RANTES), which mediates monocyte and eosinophil chemotaxis (54). This paradigm suggests that SARS-CoV-2-infected AECs likely use similar chemokine-dependent mechanisms to recruit circulating monocytes, which then differentiate into macrophages and contribute to the inflammatory response. Furthermore, SARS-CoV-2 disrupts epithelium-macrophage communication through its impact on epithelial mitochondrial function, creating a cascade that exacerbates pulmonary inflammation. Virus infection of AECs induces excessive mitochondrial fission, inhibits mitochondrial degradation, and disrupts the mitochondrial calcium uniporter. Phagocytosis of these infected epithelial cells by AMs activates the reactive oxygen species (ROS)-hypoxia-inducible factor 1α pathway, further amplifying pulmonary inflammation. Infected macrophages subsequently release large amounts of IFNs into the circulation, which upregulates mitochondrial interferon alpha-inducible protein 27 (IFI27) and impairs energy metabolism in immune cells. This cascade ultimately promotes immune dysfunction and enhances viral persistence in patients with COVID-19 (55).

Following this dysregulated communication network, abnormal regulation of the PD-1/PD-L1 immune checkpoint axis further may contribute to disease pathogenesis. PD-L1, a ligand of PD-1 and a key regulator of immune homeostasis, is significantly upregulated in SARS-CoV-2-infected epithelial cells. The studies have shown that Under normal conditions, this interaction might suppress immune activation (56). Intriguingly, studies on RSV infection have demonstrated that infected epithelial cells lose the ability to suppress monocyte activation despite upregulating PD-L1 expression (57). In COVID-19, plasma levels of pro-inflammatory cytokines and chemokines, including granulocyte-macrophage colony-stimulating factor (GM-CSF), IL-18, CCL2, CXCL10, and osteopontin, which are primarily secreted by monocytes, are markedly elevated (58). This suggests that SARS-CoV-2 infection may induce a currently unidentified mechanism that overrides PD-L1-mediated immune suppression, thereby enabling monocyte activation and sustained pro-inflammatory cytokine release, which drives the immunopathogenesis of COVID-19.

## Epithelium/dendritic cell communication

The respiratory system is continuously exposed to environmental stimuli, including pathogens, allergens, and pollutants. As the first line of defence, the airway epithelium functions as a physical barrier and supports immune surveillance. Dendritic cells (DCs), professional antigen-presenting cells strategically localized in the airway mucosa, are essential for initiating and regulating immune responses (59, 60). Therefore, communication between the airway epithelium and DCs is critical for respiratory homeostasis. Airway mucosal DCs are primarily divided into plasmacytoid DCs and conventional DCs, which differ in their origin, phenotypic markers, and function. Epithelial-DC crosstalk occurs through direct cell-cell contact, soluble mediators, and EVs (61, 62).

As key sensors of viral invasion, DCs rely on epithelial-derived signals to mount antiviral immunity (63, 64). During SARS-CoV-2 infection, the virus first invades nasal and pulmonary epithelial cells via ACE2, triggering pyroptosis, a programmed cell death pathway that releases damage-associated molecular patterns (DAMPs), PAMPs, and progeny virions. These molecules activate the innate immune response and recruit immune cells, including DCs, to lung tissues (36, 65, 66). Subsequently, activated DCs engage in reciprocal communication with epithelial cells, presenting viral antigens migrating to draining lymph nodes to activate naïve T cells (67, 68). This process leads to the activation of CD4+ helper T cells and CD8+ cytotoxic T cells, which are essential for viral clearance. In parallel, DCs secrete cytokines, such as IFNs, tumor necrosis factor (TNF), and interleukins (ILs), to orchestrate inflammatory responses and recruit additional immune cells to amplify the antiviral defence (68, 69).

**Figure 4:**
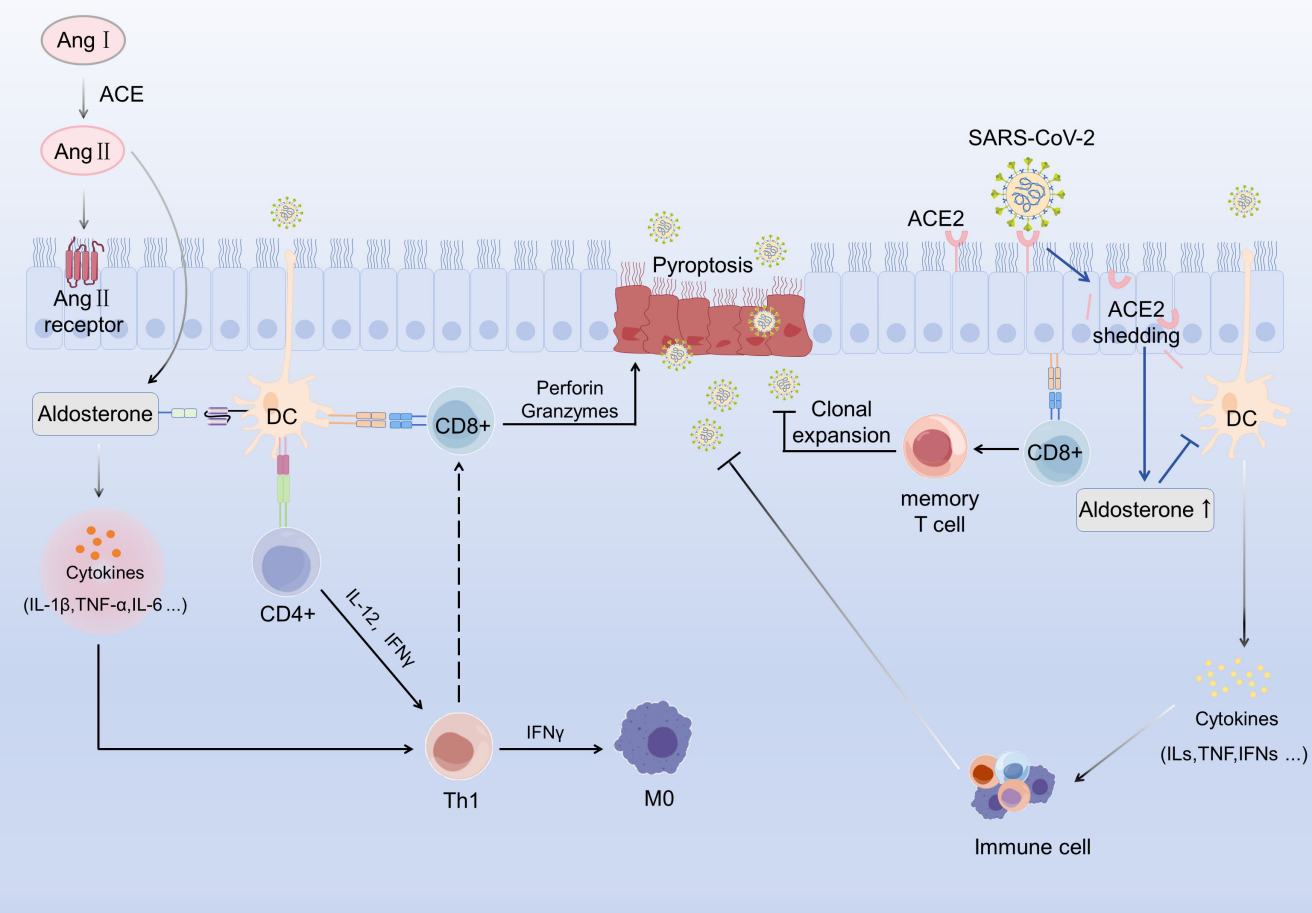
Schematic illustration of epithelium-dendritic cell (DC) communication regulated by the renin-angiotensin system (RAS) and SARS-CoV-2 infection, and its linkage to downstream immune activation and epithelial cell pyroptosis. Angiotensin I (Ang I) is converted to angiotensin II (Ang II) via angiotensinconverting enzyme (ACE); Ang II then engages its receptor on epithelial cells, triggering aldosterone secretion. Aldosterone induces the release of proinflammatory cytokines (e.g., IL-1β, TNF-α, IL-6), which orchestrate the functional polarization of DCs. Activated DCs interact with CD4+ T cells to drive their differentiation into T helper 1 (Th1) cells, which secrete IL-12 and interferon-γ (IFN-γ)—the latter acts on naive macrophages (M0) to modulate their activation. Concurrently, DCs promote CD8+ T cell activation, and the activated CD8+ T cells mediate epithelial cell pyroptosis through the perforin/granzymes pathway. SARS-CoV-2 binds to angiotensin-converting enzyme 2 (ACE2) on epithelial cells, inducing ACE2 shedding; this process also modulates aldosterone levels, which in turn reshapes DC function. DCs further drive clonal expansion of CD8+ T cells, leading to the generation of memory CD8+ T cells. Meanwhile, recruited immune cells secrete a panel of cytokines (e.g., interleukins, TNF, interferons) that participate in the immune response elicited by SARS-CoV-2 infection.

ACE2 also serves as a key regulator of the renin-angiotensin-aldosterone system (RAAS) through its interaction with angiotensin II (Ang II) (70). Mechanistically, the SARS-CoV-2 spike (S) protein bind to ACE2, facilitated by TMPRSS2, which induces ACE2 shedding, disrupts RAAS homeostasis, and ultimately leads to aldosterone overproduction (71, 72).This RAAS imbalance directly influences DC function (73). Aldosterone binds to mineralocorticoid receptors on DCs, promoting pro inflammatory cytokine secretion (71, 74, 75). Simultaneously, Ang II accumulation activates extracellular signal-regulated kinase phosphorylation, leading to increased IL-6 and TNF-α secretion (76). In patients with COVID-19, elevated Ang II levels are associated with increased aldosterone secretion and heightened IL-6 production (77, 78). Furthermore, Ang II enhances DC migration, maturation, and antigen-presenting capacity, thereby amplifying Th1 cell-mediated immune responses (79–81) (**Figure 4**).

In summary, epithelial-DC crosstalk may be a central regulatory axis in respiratory immunity during SARS-CoV-2 infection. This shifts epithelial-DC communication toward a pro-inflammatory state, underscoring its dual role in being essential for viral clearance yet a major contributor to COVID-19-associated immunopathology.

## Epithelium/neutrophil communication

Neutrophils are the most abundant leukocytes in peripheral blood and are among the first immune cells to infiltrate sites of infection. They play a pivotal role in host defence against bacterial and viral pathogens by phagocytosing microbes, releasing granule contents such as antimicrobial peptides and proteases, and producing ROS and pro-inflammatory cytokines (82, 83). Upon viral infection, airway epithelial cells secrete chemokines, including IL-8 and granulocyte colony-stimulating factor (G-CSF), that recruit neutrophils to the infection site (84, 85). Once recruited, neutrophils become activated and deploy antimicrobial mechanisms.

**Figure 5:**
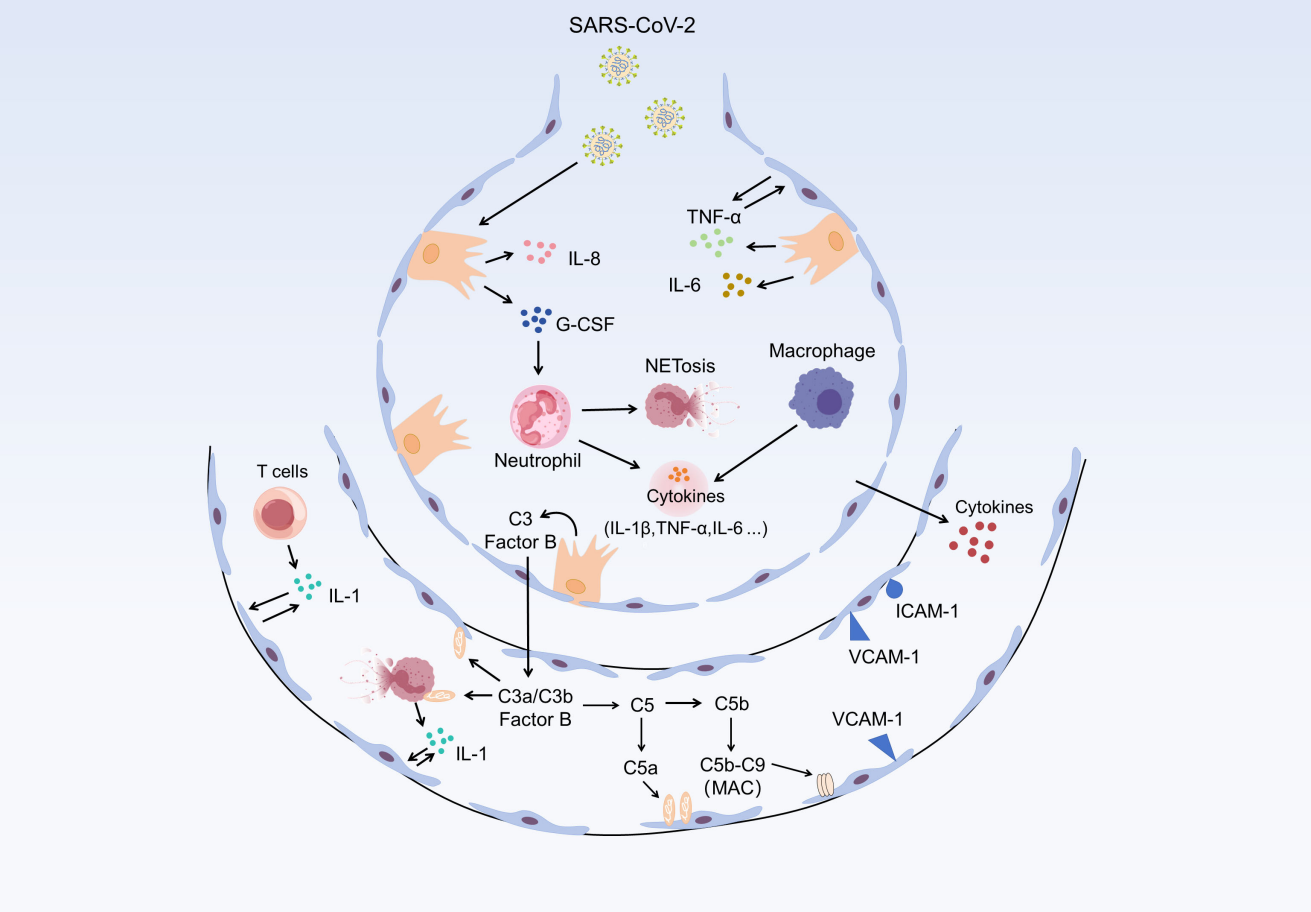
Schematic depiction of epithelium-neutrophil communication during SARS-CoV-2 infection. SARS-CoV-2 engages epithelial cells, which subsequently secrete a panel of proinflammatory mediators including interleukin-8 (IL-8), granulocyte colony-stimulating factor (G-CSF), tumor necrosis factor-α (TNF-α), and interleukin-6 (IL-6). These factors orchestrate the recruitment and activation of neutrophils. Activated neutrophils undergo NETosis (neutrophil extracellular trap formation) and release cytokines (e.g., IL-1β, TNF-α, IL-6), which further recruit macrophages; macrophages in turn secrete additional cytokines to amplify the inflammatory signaling. Concurrently, epithelial cell-associated complement components (C3, Factor B) are activated, leading to the generation of C3a/C3b and C5 fragments. C5 is cleaved into C5a (a potent chemoattractant) and C5b; C5b subsequently assembles with downstream complement components to form the membrane attack complex (MAC, C5b-C9), which mediates cellular injury. Additionally, C3a and C5a contribute to the secretion of proinflammatory factors (e.g., IL-1) to sustain the inflammatory microenvironment. Epithelial cells upregulate the expression of intercellular adhesion molecule 1 (ICAM-1) and vascular cell adhesion molecule 1 (VCAM-1), which facilitate the adhesion and recruitment of immune cells such as T cells. Crosstalk between T cells and epithelial cells (e.g., via IL-1 signaling) also participates in the regulation of the local inflammatory milieu. Collectively, this epithelium-neutrophil communication network—coupled with complement activation and multi-cell crosstalk—drives the excessive inflammatory response during SARS-CoV-2 infection.

During SARS-CoV-2 infection, crosstalk between airway epithelial cells and neutrophils has emerged as a critical determinant of disease outcome. This interaction is multifaceted and modulates both infection progression and tissue homeostasis. Histological analyses of explanted lung tissues from patients with COVID-19 reveal extensive epithelial pathology, including epithelial detachment and necrosis, associated with robust neutrophil infiltration and activation. These findings confirm that neutrophils are actively involved in the host response to SARS-CoV-2 (37, 86). The mechanisms of epithelial-neutrophil communication can be divided into three main steps: first, pro-inflammatory signal amplification; infection of RECs induces neutrophil recruitment, which in turn promotes the release of pro-inflammatory cytokines, such as IFN-γ, IL-1β, IL-6, and TNF-α. These neutrophil-derived mediators disrupt epithelial barrier integrity and increase permeability (87, 88). The coexistence of neutrophils with conducting airway epithelia establishes a polarized pro-inflammatory niche that accelerates epithelial barrier breakdown. This disruption further facilitates infection of deeper epithelial layers, including basal stem cells that are essential for epithelial repair (37). Second, NET-mediated effects: neutrophils release NETs, which are web-like structures composed of chromatin, histones, and antimicrobial proteins. While NETs can immobilize and eliminate pathogens, their excessive formation and impaired clearance contribute to tissue damage, including alveolar epithelial injury and vascular leakage (38, 89). Neutrophils also secrete IL-1β, TNF-α, and IL-6, further modulating the magnitude and duration of the immune response. Maintaining an appropriate balance between pro-inflammatory and anti-inflammatory signals is crucial for promoting viral clearance while avoiding immunopathology. Third, direct cell-cell contact: airway epithelial cells and neutrophils engage in physical interactions that regulate neutrophil activation, apoptosis, and the resolution of inflammation (**Figure 5**).

Overall, communication between airway epithelial cells and neutrophils is a central feature of immune response to SARS-CoV-2. While essential for early defence, excessive neutrophil recruitment and activation might exacerbate immune-mediated tissue damage, impair viral clearance, and exacerbate disease severity. A detailed understanding of epithelial-neutrophil crosstalk may inform therapeutic strategies aimed at reducing COVID-19 severity by modulating neutrophil activity without compromising antiviral defence.

## Epithelium/lymphocyte communication

Cellular crosstalk in the respiratory system is indispensable for mounting effective adaptive immunity against SARS-CoV-2. This communication orchestrates the transition from innate to adaptive responses, while its dysregulation drives immunopathological damage. Several studies have shown that during infection, RECs initiate innate immune responses through the secretion of cytokines, chemokines, and type III IFNs, which recruit and activate lymphocytes to restrict viral replication and dissemination (90, 91). Bronchial epithelial cells primarily secrete pro-inflammatory cytokines (such as IL-6 and IL-1β) and chemokines (including CXCL10 and CCL2), which bind to receptors (such as CCR5 and CXCR3) on lymphocytes to guide their migration (92–94). By contrast, AECs preferentially produce type III IFNs that induce IFN-stimulated genes (e.g., MX1 and OAS) in lymphocytes, thereby enhancing their antiviral capacity (95, 96). In addition to cytokine release, epithelial cells present viral antigens via MHC molecules: MHC class I presentation activates CD8+ cytotoxic T cells, while MHC class II presentation activates CD4+ helper T cells. Activated lymphocytes, in turn, secrete IFN-γ and IL-4 to regulate epithelial inflammation and barrier repair, establishing a bidirectional communication loop (97, 98).

Programmed cell death of SARS-CoV-2-infected AECs further shapes immune responses. Necroptosis of AECs releases pro-inflammatory DAMPs, including high-mobility group box 1, which amplify inflammation and exacerbate lung injury. CD8+ CTLs induce Fas-Fas ligand (FasL)-mediated apoptosis of infected epithelial cells, a process that eliminates viral reservoirs while releasing DAMPs that reinforce immune activation (34, 99). In parallel, alveolar and bronchial epithelial cells secrete B cell-stimulatory molecules, such as B cell-activating factor and a proliferation-inducing ligand (APRIL), which drive B cell activation and promote local IgM and IgA production by airway-resident B cells (100, 101).

Dysregulated epithelium-lymphocyte communication contributes to two major pathological outcomes. First, excessive secretion of cytokines, such as IL-6, and chemokines, such as CXCL10, by infected epithelial cells promotes a cytokine storm. SARS-CoV-2-infected AECs undergo apoptosis and necroptosis with dual consequences. Fas-FasL-mediated apoptosis restricts infection by eliminating virus-infected cells, whereas inflammatory cell death pathways (such as necroptosis and pyroptosis) release large quantities of DAMPs that fuel immune hyperactivation, lung destruction, multi-organ damage, and increased mortality. Clinical data show IL-6 levels in bronchoalveolar lavage fluid from patients with severe COVID-19 are 8–10 times higher than in mild cases, correlating with lymphocyte apoptosis and dysfunction (102, 103). Importantly, epithelial signals can also mediate a T cell-independent antibody response, in which B cells are activated without CD4+ T cells. This rapid antibody production contributes to antiviral protection and may play a role in preventing reinfection by SARS-CoV-2.

**Figure 6:**
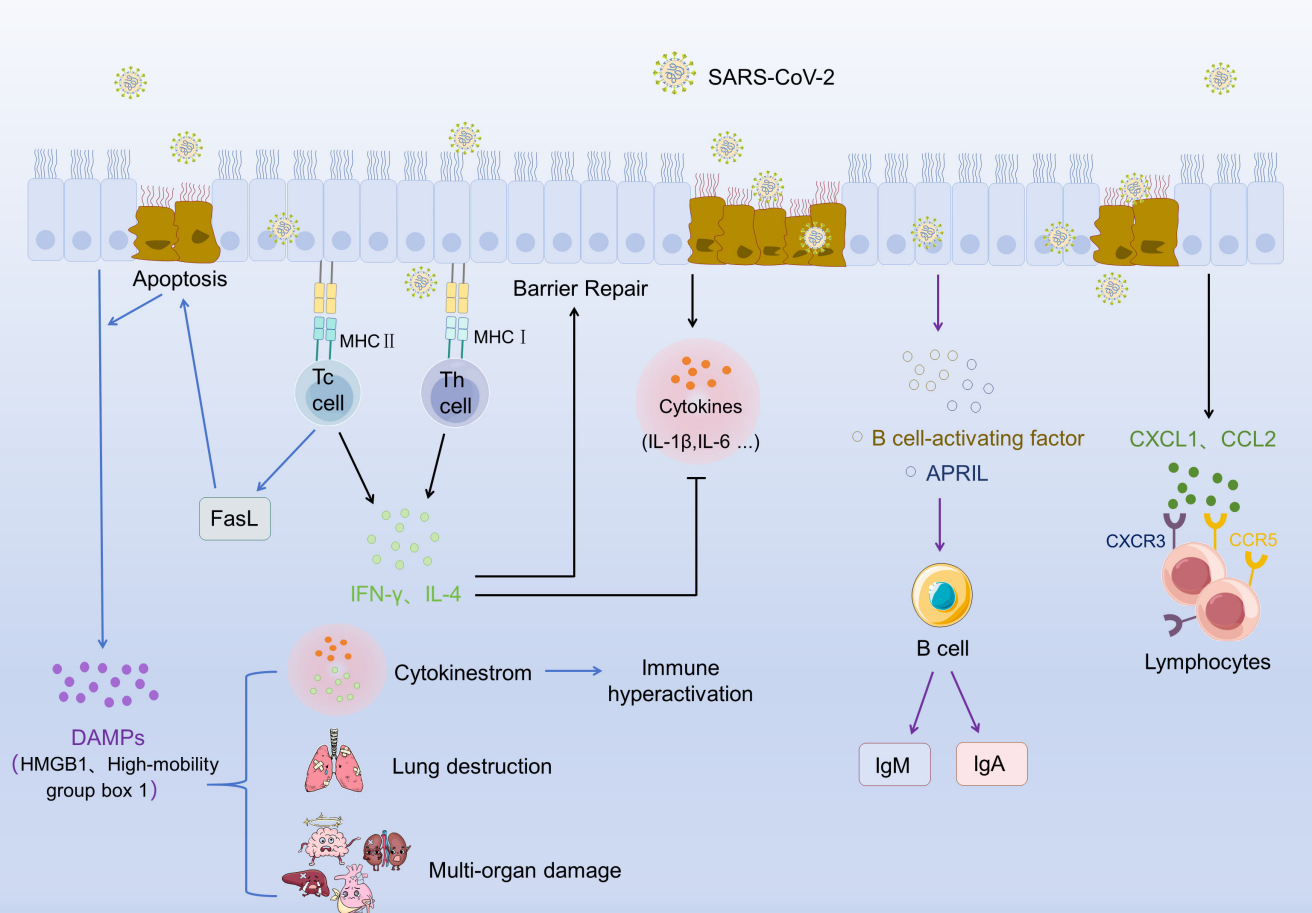
Schematic illustration of epithelium-lymphocyte communication during SARS-CoV-2 infection: integrating antiviral immune responses, epithelial barrier dynamics, and immunopathogenic cascades. SARS-CoV-2 invades respiratory epithelial cells, inducing apoptosis in a subset of these cells. Apoptotic epithelial cells release damage-associated molecular patterns (DAMPs, e.g., HMGB1 [high-mobility group box 1]) and transduce apoptotic signals via Fas ligand (FasL). Cytotoxic T (Tc) cells recognize epithelial cell-derived antigens through major histocompatibility complex class II (MHC II) molecules, mediating targeted immune surveillance against infected epithelial cells. Helper T (Th) cells interact with epithelial cells via MHC class I (MHC I) molecules, secreting cytokines (IFN-γ, IL-4) that exert dual effects: these factors promote epithelial barrier repair, while also driving epithelial cells to secrete proinflammatory cytokines (e.g., IL-1β, IL-6). Excessive accumulation of proinflammatory cytokines triggers a cytokine storm, leading to immune hyperactivation, subsequent lung destruction, and multi-organ damage. Epithelial cells secrete B cell-activating factor (BAFF) and a proliferation-inducing ligand (APRIL), which activate B cells to differentiate and produce antiviral antibodies (IgM, IgA). Additionally, epithelial-derived chemokines (CXCL1, CCL2) recruit lymphocytes by engaging their cognate receptors (CXCR3, CCR5), thereby modulating the composition of local immune infiltrates. Epithelium-lymphocyte communication during SARS-CoV-2 infection orchestrates a balance between protective antiviral defense/barrier homeostasis and dysregulated immunity that underlies pathological tissue injury.

Thus, epithelium-lymphocyte crosstalk represents a central regulatory hub of respiratory adaptive immunity during SARS-CoV-2 infection. Epithelial cells orchestrate lymphocyte recruitment and activation via cytokines, chemokines, and antigen presentation, while lymphocytes reciprocally regulate epithelial function. However, when dysregulated, this crosstalk underlies cytokine storms and inflammatory cell death, might make it a key driver of COVID-19 immunopathology. Targeting this communication network may offer therapeutic opportunities to balance antiviral defence with immune homeostasis (**Figure 6**).

## Communication back to epithelial cells

In addition to transmitting signals to other cell populations, RECs receive feedback signals from lung-resident immune and stromal cells. This feedback is critical for repairing epithelium damage during the resolution phase of viral infections. Growth factors are central reparative signals, but their effects depend on timing and may increase the risk of worsening infections. As described earlier, GM-CSF is secreted by AECs during viral infection and is induced by TNF-α produced by activated AMs (104, 105). GM-CSF promotes proliferation of AT2 cells, thereby driving epithelial repair and restoring barrier function (106). Keratinocyte growth factor (KGF) inhibits AEC differentiation and enhances AT2 cell proliferation to protect against alveolar damage, which helps maintain homeostasis (107, 108). However, epithelial-derived IL-1β stimulates fibroblasts to produce KGF, and this fibroblast-derived KGF increases the susceptibility of AT2 cells to viral infection, likely by upregulating viral entry receptors. The precise mechanism underlying this regulatory cascade remains incompletely understood (109). The dual effects of growth factors are closely linked to their timing. During the active phase of viral replication, overproduction of growth factors by epithelial cells and fibroblasts may enhance viral invasion and worsen disease. In contrast, at later stages, when viral load decreases, growth factors primarily stimulate AT2 cell proliferation and promote epithelial repair. These opposing effects underscore the importance of timing in determining whether growth factors facilitate recovery or contribute to pathogenesis.

Macrophages activated by SARS-CoV-2-infected epithelial cells also deliver feedback signals that directly impair epithelial function. Specifically, they inhibit Na^+^/K^+^-ATPase activity, a key pump mediating epithelial fluid reabsorption (110, 111). Inhibition of this pump reduces alveolar fluid clearance and promotes fluid accumulation, thereby contributing to pulmonary pathology. In contrast, immune cells can provide positive feedback that amplifies epithelial antiviral responses. For example, type I IFN signaling from monocytes stimulates bronchial epithelial cells to produce CXCL10 during rhinovirus infection, enhancing local immune defence.

Together, the feedback signals received by epithelial cells create a dynamic “repair-pathogenesis-synergy” regulatory network. Growth factors act as core reparative signals but can also increase infection risk depending on timing; macrophage-derived signals may directly impair epithelial function and promote pathogenesis, and immune cell-derived synergistic signals strengthen antiviral defences. The balance of these opposing signals may determine the progression and outcome of respiratory viral infections.

## Discussion

RECs are pivotal mediators of immune crosstalk during SARS-CoV-2 infection, functioning as both primary viral targets and core regulators of the balance between antiviral immunity and immunopathology (37, 43, 55). Through the secretion of cytokine and chemokine, antigen presentation, regulation of the PD-L1 checkpoint, and modulation of the RAAS, RECs engage in bidirectional interactions with macrophages, DCs, neutrophils, and lymphocytes, thereby exerting dual effects on COVID-19 progression (8, 56, 71). Physiologically, REC-derived type III interferons, GM-CSF, and MHC molecule-mediated antigen presentation promote viral clearance and epithelial repair (95, 97, 106). However, dysregulated crosstalk such as excessive IL-6 and TNF-α secretion, M1 macrophage polarization, NET formation, and ACE2 shedding-induced RAAS imbalance—triggers cytokine storms, epithelial barrier damage, and ALI (30, 37, 48, 74). Feedback signals from immune cells further shape this network: growth factors like KGF support repair but may enhance viral susceptibility depending on the temporal window of expression (107, 109). Meanwhile, activated macrophages inhibit alveolar fluid reabsorption via Na^+^/K^+^-ATPase suppression, highlighting the complexity of REC-mediated immune regulation (110, 111).

Emerging evidence increasingly supports the view that respiratory epithelial cells (RECs) are not merely passive targets of SARS-CoV-2 infection, but rather function as central organizers of the local immune landscape. In this context, REC–immune communication is better conceptualized as an integrated and dynamic network than as a series of isolated pairwise interactions with individual immune-cell subsets (37, 43, 55). At the onset of infection, RECs act as frontline sentinels that detect viral invasion and initiate alarm programs through innate immune sensing, type I/III interferon production, and chemokine release, thereby shaping the recruitment and functional priming of macrophages, dendritic cells (DCs), neutrophils, and lymphocytes (29, 65, 68, 95). As infection progresses, this epithelial alarm state becomes coupled to additional regulatory layers, including DAMP release, antigen presentation, PD-L1-associated checkpoint modulation, and ACE2/RAAS disequilibrium, allowing RECs to influence both antiviral immunity and inflammatory escalation (33, 56, 71, 74). Thus, the epithelial compartment occupies a strategically central position at the interface between viral sensing, immune orchestration, and tissue injury.

Within this REC-centered framework, the different immune populations discussed in this review can be viewed as functionally interconnected modules rather than parallel and independent axes. Macrophages amplify inflammatory circuits and influence viral persistence or resolution (48, 53); DCs link epithelial sensing to antigen presentation and adaptive immune priming (67, 68, 73); neutrophils reinforce inflammatory injury through cytokine release, protease activity, and NET formation (37, 38, 89); and lymphocytes contribute to both viral clearance and secondary immunopathology through cytotoxicity, cytokine production, and local humoral support (97, 100, 101). Importantly, these interactions are not unidirectional. Feedback signals derived from immune and stromal cells subsequently reshape epithelial function, influencing barrier integrity, alveolar fluid handling, metabolic fitness, regenerative responses, and, ultimately, disease trajectory (44, 46, 55, 106–108, 111). Taken together, these observations support a unified model in which REC-mediated crosstalk is organized across three closely linked layers: epithelial sensing and alarm initiation, immune amplification and diversification, and repair-versus-pathology feedback. This conceptual framework may help reconcile seemingly heterogeneous findings across studies and provides a more coherent basis for understanding how epithelial–immune cooperation shifts from protective antiviral defense to maladaptive immunopathology during COVID-19.

Although longitudinal and stage-resolved evidence remains incomplete, the current literature supports a useful conceptual model in which REC–immune crosstalk evolves across distinct yet overlapping phases of SARS-CoV-2 infection. In the early phase, RECs primarily function as viral sensors and first responders. Viral entry through ACE2 and associated host proteases activates epithelial innate immune programs, leading to the induction of type I and particularly type III interferons, together with chemokines that initiate myeloid- and lymphoid-cell recruitment (20, 28, 29, 43, 95). At this stage, epithelial signaling is predominantly protective, as it contributes to local viral restriction, immune priming, and maintenance of an antiviral tissue state (65, 69, 96). This phase may therefore represent a critical window during which REC-derived signals are biased toward host defense rather than tissue damage.

With ongoing viral replication and epithelial injury, REC–immune crosstalk may then transition into an inflammatory amplification phase. During this stage, continued release of DAMPs, pro-inflammatory cytokines, chemokines, and procoagulant mediators intensifies communication with macrophages, DCs, neutrophils, and activated lymphocytes (10, 33, 36, 37, 53). Mechanistically, pathways that are initially adaptive may become maladaptive when sustained or excessive, promoting macrophage inflammatory activation, IL-6/TNF-α-driven cytokine escalation, RAAS imbalance, NET-associated epithelial and endothelial injury, and progressive barrier dysfunction (30, 37, 48, 74, 77, 89). In this setting, RECs no longer simply coordinate antiviral defense, but may become active propagators of feedforward inflammatory damage.

As viral burden declines, the system may enter a resolution and repair phase, although in some patients this trajectory may be replaced by persistent epithelial dysfunction and chronic inflammatory signaling. In this later stage, the balance shifts toward epithelial regeneration, AT2-cell proliferation, barrier restoration, and tissue remodeling, with increasing influence from macrophage-, lymphocyte-, and stromal-derived feedback signals (35, 106–109). Notably, the biological effects of these pathways are highly time-dependent: signals that promote epithelial recovery during convalescence may be detrimental if activated prematurely during active viral replication (107–109). Conversely, unresolved macrophage-derived dysfunction, altered epithelial metabolism, and persistent barrier damage may contribute to prolonged respiratory abnormalities and potentially to long COVID-related sequelae (55, 111). Therefore, incorporation of a temporal perspective is essential not only for interpreting REC–immune biology, but also for informing stage-specific therapeutic strategies, including early reinforcement of antiviral defense, subsequent control of injurious inflammatory amplification, and later support of epithelial repair and immune homeostasis (30, 37, 71, 95, 106).

Despite these emerging mechanistic and conceptual advances, critical gaps remain in our understanding of REC–immune crosstalk during SARS-CoV-2 infection. These gaps include the ambiguous role of PD-L1 upregulation in infected RECs—where immune suppression is expected but monocyte activation remains elevated, the discrepancies in SARS-CoV-2 replication efficiency between M1 and M2 macrophages across different culture systems (48, 49, 51), and the inconsistent correlations between type III interferon levels and disease severity (29, 69). RECs across the respiratory tract, such as nasal epithelial cells, bronchial secretory cells, and AT2 cells display distinct gene expression and functional profiles (43), but the unique contributions of each subpopulation to antiviral immunity versus immunopathology remain undefined. For example, it is unclear whether AT2 cells and bronchial epithelial cells differ in their capacity to regulate macrophage polarization or lymphocyte activation. 2D *in vitro* models and immune cell-deficient organoids fail to recapitulate *in vivo* respiratory microenvironments (22, 27), animal models lack human-like ACE2 expression and comorbidity diversity (14, 39), and most clinical studies are cross-sectional with small sample sizes (7, 30). Notably, SARS-CoV-2 variants (e.g., JN.1, LP.8.1) modulate REC-immune crosstalk. However, the functional heterogeneity of RECs across respiratory tract regions, and the role of persistent REC dysfunction in long COVID pathogenesis remain unclear (7, 34, 43).

From a translational perspective, targeting REC–immune crosstalk pathways offers several promising therapeutic opportunities: IL-6 neutralization with tocilizumab mitigates cytokine storm (30, 102), NET inhibitors reduce epithelial injury (37, 38), and ACE2 agonists restore RAAS balance to suppress DC hyperactivation (71, 77). Therapeutic strategies must be stage-specific—enhancing antiviral immunity in the early phase, inhibiting excessive inflammation acutely, and promoting repair in late phases—while accounting for patient genetic diversity and viral variant characteristics (95). Future research should prioritize 3D immune-competent airway organoids, humanized comorbidity models, and large-scale prospective clinical studies to clarify the dynamic mechanisms of REC-immune crosstalk, resolve conflicting findings, and inform the precision therapies. Although longitudinal evidence remains incomplete, integrating a stage-dependent view of REC–immune crosstalk will be essential for clarifying COVID-19 pathogenesis and for guiding temporally stratified therapeutic strategies aimed at preserving antiviral immunity while limiting immunopathology and promoting epithelial recovery (30, 37, 43, 95, 106).

## Conclusions

Recent studies dissecting the crosstalk between SARS-CoV-2-infected RECs and immune cells have advanced our understanding of COVID-19 pathogenesis by clarifying how RECs shape immune cell behaviour and how immune feedback influences epithelial function. As the primary targets of SARS-CoV-2 (43), RECs orchestrate bidirectional interactions with macrophages, DCs, neutrophils, and lymphocytes through cytokine signaling, antigen presentation via MHC molecules, RAAS dysregulation, and checkpoint molecules such as PD-L1. These interactions exert dual effects: they can promote viral clearance, for example through type III IFNs enhancing lymphocyte antiviral activity, via MHC molecule-mediated antigen presentation to activate specific T/B cell responses, or drive immunopathology, such as through excessive IL-6 secretion triggering cytokine storms (30).

REC-derived signals influence macrophage polarization, favouring proinflammatory M1 states over protective M2 states; ACE2 shedding amplifies DC-mediated inflammation through Ang II/aldosterone signaling; and NETs induced by REC chemokines disrupt epithelial barriers (37). Meanwhile, the regulatory role of immune cell-derived growth factors (e.g., GM-CSF and KGF) in epithelial repair via feedback signals provides additional targets for optimizing therapeutic strategies. Together, these findings highlight targeting pathological crosstalk, such as IL-6 neutralization with tocilizumab, or enhancing protective interactions, such as ACE2 agonists, to restore RAAS balance as therapeutic options to control disease severity by modulating REC-immune interactions.

Overall, elucidating REC-immune communication dynamics is critical for developing targeted interventions to mitigate severe COVID-19. Leveraging this crosstalk offers a strategy to strengthen antiviral immunity while minimizing immunopathology, providing a framework for therapeutic innovation.

## Glossary

AECs: alveolar epithelial cells
AT1: type 1 alveolar epithelial cells
AT2: type 2 alveolar epithelial cells
RECs: respiratory epithelia cells
ARDS: acute respiratory distress syndrome
RSV: respiratory syncytial virus
WHO: World Health Organization
GISRS: Global Influenza Surveillance and Response System
TLR: Toll-like receptor
CXCL10: CXC chemokine ligand 10
TF: tissue factor
EVs: extracellular vesicles
SARS-CoV: severe acute respiratory syndrome coronavirus
COVID-19: coronavirus disease 2019
ACE2: angiotensin-converting enzyme 2
TMPRSS2: transmembrane serine protease 2
HBECs: human bronchial epithelial cells
AMs: alveolar macrophages
PAMPs: pathogen-associated molecular patterns
RANTES: regulated on activation, normal T cell expressed and secreted
GM-CSF: granulocyte-macrophage colony-stimulating factor
DCs: Dendritic cells
DAMPs: damage-associated molecular patterns
TNF: tumor necrosis factor
ILs: interleukins
RAAS: renin-angiotensin-aldosterone system
G-CSF: granulocyte colony-stimulating factor
NET: neutrophil extracellular trap
FasL: Fas-Fas ligand
APRIL: a proliferation-inducing ligand
KGF: Keratinocyte growth factor
KGF: Keratinocyte growth factor
